# Supplementary material for: Pressure-Driven Perfusion System to Control, Multiplex and Recirculate Cell Culture Medium for Organs-on-Chips
Source: Micromachines (Basel). 2022 Aug 20;13(8):1359. doi: 10.3390/mi13081359 (PMC9416133; doi:10.3390/mi13081359)
Supplement: Supplementary file 1 [file micromachines-13-01359-s001.zip › Suplnfo/SupI_PID.pdf]

Supplementary information

Pressure-driven perfusion system to control, multiplex and recirculate cell culture medium for Organs-on-Chips

Mees N. S. de Graaf[1], Aisen Vivas[2,3], Andries D. van der Meer[2], Christine L. Mummery[1,2], Valeria V. Orlova[1*]

[1] Department of Anatomy and Embryology, Leiden University Medical Center, Einthovenweg 20, 2333ZC Leiden, the Netherlands

[2] Applied Stem Cell Technologies, 7500AE Enschede, University of Twente, The Netherlands

[3] BIOS Lab on a Chip Group, MESA+ Institute for Nanotechnology, Technical Medical Centre, Max Planck Institute for Complex Fluid Dynamics, University of Twente, 7500AE Enschede, The Netherlands

*Corresponding author e-mail address: v.orlova@lumc.nl

Supplementary Figures

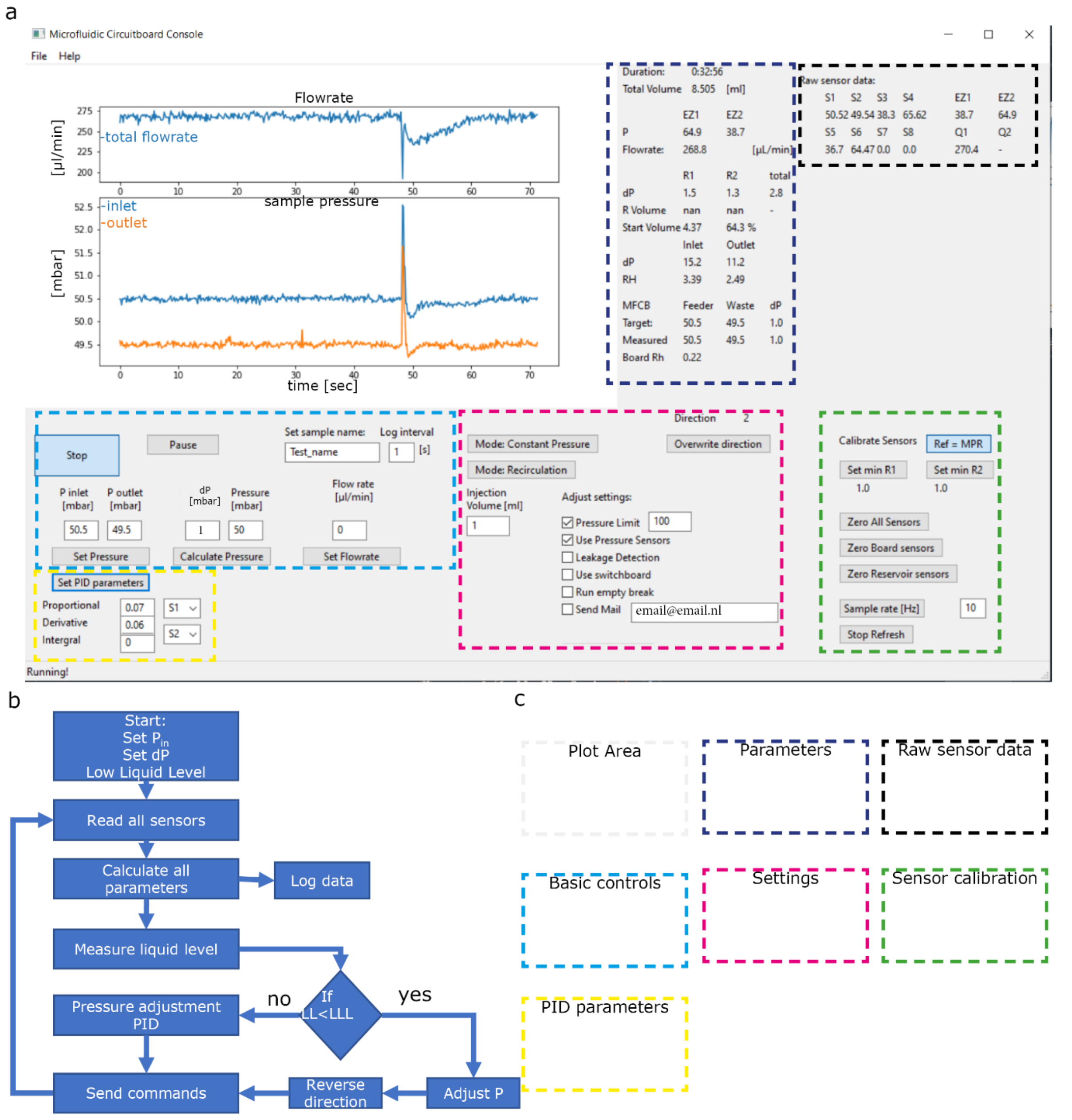

Figure. S1 Software overview (a) screen shot of the custom software showing controlling buttons and parameters plots(b) Feedback loop of the software. It starts with reading all sensors and calculate the pressure difference between the inlet and outlet, internal pressure and liquid levels. The PID-controller will adjust the commands to reach the setpoints. (c) Overview of all functions and parameters, explanation in table s1.

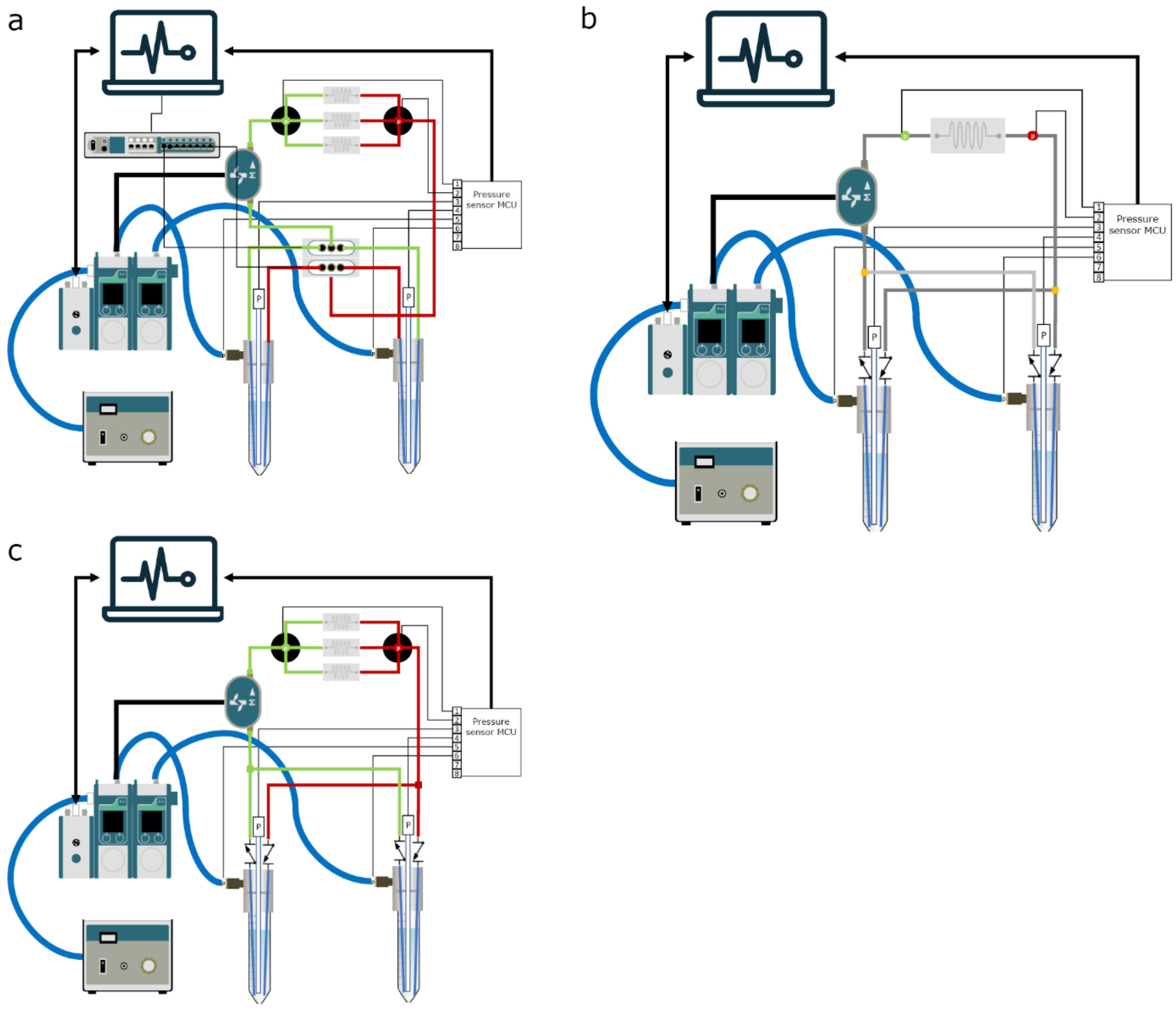

Figure. S2 Examples of alternative fluidic circuits (a) recirculation using actively controlled 3/2 valves(Fluigent)(b)Single OoC perfusion,(c) multiplexing using of the shelf manifold splitters.

Table S1. Software functionality

Parameters	Info:
Duration	Total experimental time
Total Volume	Total displaced volume
P	Pressure at controller (filtered)
Flowrate	Total flowrate (filtered)
dP R1 R2	Measured P-head (filtered)
R volume	Calculate Volume
Start volume	Start volume, % measured at present
inlet dP	dP inlet Reservoir to Feeder channel
outlet dP	dP Waste-channel to outlet Reservoir
MFCB	Sample parameters
Target Pressure	target difference pressure
Measured Pressure	Measured pressure (difference)(filtered)
Board Rh	Sample resistance(filtered)
Raw sensor data	Unfiltered sensor output
**Basic control**	
start/stop	Start / stop experiments
Pause	Sends no commands, keeps logfile alive
P inlet/P outlet	Set target inlet/outlet
Set pressure	Sends input to controller
Shear/dp/Pressure	set target pressure difference and internal pressure
calculate pressure	Sends input to controller
Set flowrate	Set flowrate
Set sample name	Logfile name
log interval	sets log frequency in seconds
**PID parameters**	
Set PID parameters	Adjust parameters
Proportional	P-parameter
Derivative	D-parameter
Integral	I-parameter
Settings	
Mode: Constant Pressure / Constant flowrate	Change process variable
Mode: Recirculation / Injection	Continuous recirculation, Injection target volume with recirculation
injection volume	Target injection
overwrite direction	force direction switch
Pressure limit	Limits pressure to protect sample
Use pressure sensors	Use manufactures PID loop
Leakage detection	Switch off when volume is below 85% of start volume
Use switchboard	sends 3/2 switch commands
Run empty break	If flow>0 break
send mail	Sends emails with Situation Reports or leakage/empty break
**Sensor calibration**	
Ref MPR/EZ	Calculate reservoir dP with EZ command or pressure sensor
Set min R1/R2	set low liquid level
Zero all sensors	auto zero all sensors
Zero board sensors	auto zero board sensors
Zero reservoir sensors	auto zero reservoir sensors
Sample rate	Change frequency of algorithm
Stop refresh	Pauses algorithm

Table S2. Channel dimensions FCB

Dynamic Viscosity	Cell culture medium @ 37 °C, 2% serum[1]			7.90E-04 [Pa S]
	Length [m]	Width [m]	Height [m]	R_h [$Pa\ s\ m^{-3}$]
Feeder channel	0.2	0.0025	0.002	1.90E+08
Feeder loop	0.01	0.0012	0.002	4.40E+07
			Total	2.34E+08
Waste channel	0.2	0.0025	0.002	1.90E+08
waste loop	0.082	0.0035	0.002	4.33E+07
			Total	2.34E+08
Resistor calculated	0.010	5.00E-04	2.50E-04	1.77E+10
Total Chip connection				4.39E+10
Empty microfluidic channel	0.011	5.00E-04	5.00E-04	4.48E+09

a b

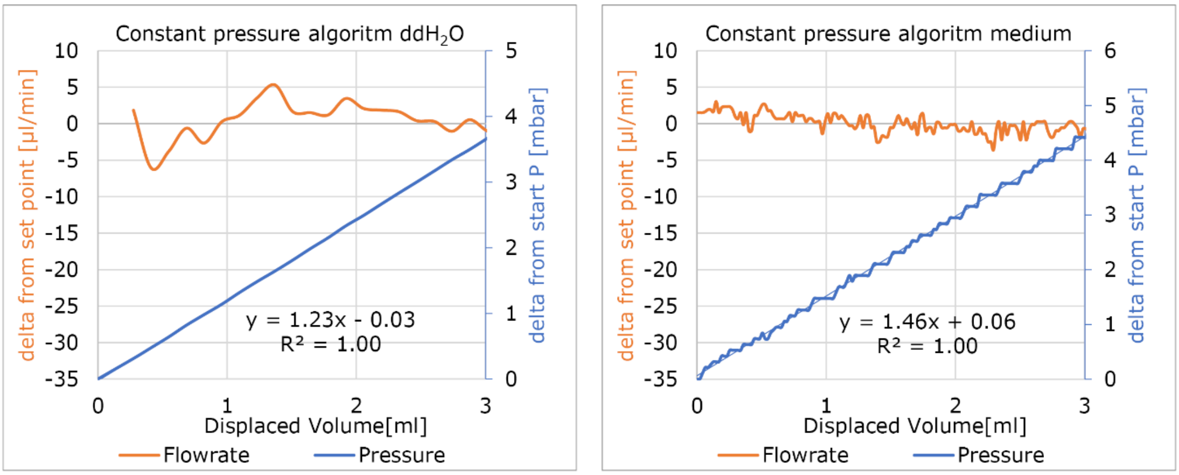

Figure. S3 Typical total pressure increase using the custom PID-controller

(a) Pressure increase per displaced ml of ddH20s. (b) Pressure increase per displaced ml of medium

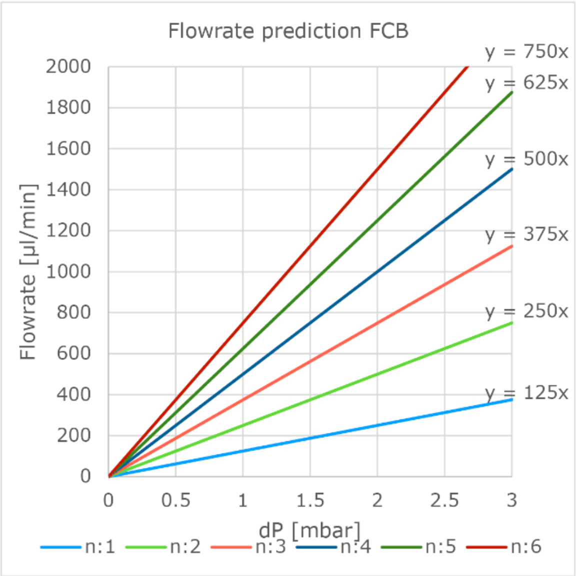

Figure. S4 Prediction of flowrates

Prediction of multiplexed microfluidic channels (1 to 6) using pressure controlled perfusion. Dimensions listed in **table s2** and EQ 5 and 6 were used to calculate this prediction.

References

1 Poon, C. Measuring the density and viscosity of culture media for optimized computational fluid dynamics analysis of in vitro devices. *J Mech Behav Biomed Mater* **126**, 105024, doi:10.1016/j.jmbbm.2021.105024 (2022).
